# Individualized treatment strategies for primary hepatic neuroendocrine carcinoma: Two case reports and literature review (A CARE-compliant case report)

**DOI:** 10.1097/MD.0000000000047242

**Published:** 2026-01-23

**Authors:** Haiyang Hu, Kai Chen, Heng Xiao, Xiang Lan

**Affiliations:** a Department of Hepatobiliary Surgery, The First Affiliated Hospital, Chongqing Medical University, Chongqing, China.

**Keywords:** case report, conversion therapy, hepatic arterial infusion chemotherapy (HAIC), primary hepatic neuroendocrine carcinoma (PHNET), transarterial chemoembolization (TACE)

## Abstract

**Rationale:**

Primary hepatic neuroendocrine carcinoma (PHNET) is an exceptionally rare malignancy with limited standardized treatment options.

**Patient concerns:**

Two patients presented with incidentally detected hepatic masses and nonspecific gastrointestinal symptoms.

**Diagnoses:**

Case 1 was diagnosed as primary hepatic neuroendocrine carcinoma (NEC, G3), and Case 2 as primary hepatic large-cell neuroendocrine carcinoma (LCNEC, G3), based on histopathology and immunohistochemistry after excluding extrahepatic origins.

**Interventions:**

Case 1 received transarterial chemoembolization (TACE), etoposide–cisplatin chemotherapy, hepatic arterial infusion chemotherapy (HAIC), and octreotide. Case 2 underwent 3 cycles of drug-eluting bead TACE (d-TACE), HAIC, and long-acting octreotide for symptomatic control of diarrhea.

**Outcomes:**

Case 1 experienced progressive disease and died of sepsis. Case 2 achieved significant tumor regression, allowing curative resection. No recurrence was observed at one-month follow-up.

**Lessons:**

The combination of d-TACE, HAIC, and octreotide may provide a potential downstaging approach for unresectable PHNET, but evidence remains preliminary and hypothesis-generating.

## 1. Introduction

Neuroendocrine tumors (NETs), formerly known as carcinoid tumors, are a heterogeneous group of neoplasms originating from peptidergic neurons and neuroendocrine cells, primarily arising in the gastrointestinal, respiratory, and genitourinary systems. The liver is a common site of metastasis for these tumors.^[1]^ Primary hepatic neuroendocrine tumor (PHNET) is exceedingly rare, accounting for only 0.3% of all NETs and 0.4% of resected primary hepatic malignancies.^[2–4]^ The origin of PHNET remains unclear and is still a matter of debate.^[5–7]^

Clinically, PHNET lacks specific symptoms, with only a minority of patients exhibiting classic carcinoid syndrome manifestations such as flushing, abdominal pain, or diarrhea.^[5]^ Consequently, 87% of patients are diagnosed at an advanced stage with multifocal liver involvement.^[6]^ Diagnosis relies on a combination of laboratory testing, imaging, and definitive histopathological and immunohistochemical confirmation.^[7]^ Surgical resection, including hepatectomy and liver transplantation, remains the preferred treatment modality.^[5,8,9]^ However, therapeutic strategies for unresectable cases with intrahepatic metastases are still under debate. Case reports have suggested potential benefits from systemic chemotherapy and transarterial chemoembolization (TACE).^[10–12]^

This study presents 2 cases of PHNET treated at our center. Drawing on the lessons learned from the first case, a more effective individualized strategy was implemented in the second case, ultimately leading to curative resection. Given the descriptive nature of this two-case report, no statistical analyses were performed. These findings may provide valuable insights for the management of future patients with unresectable PHNET.

## 2. Case reports

### 2.1. Case1

**Figure 1.**
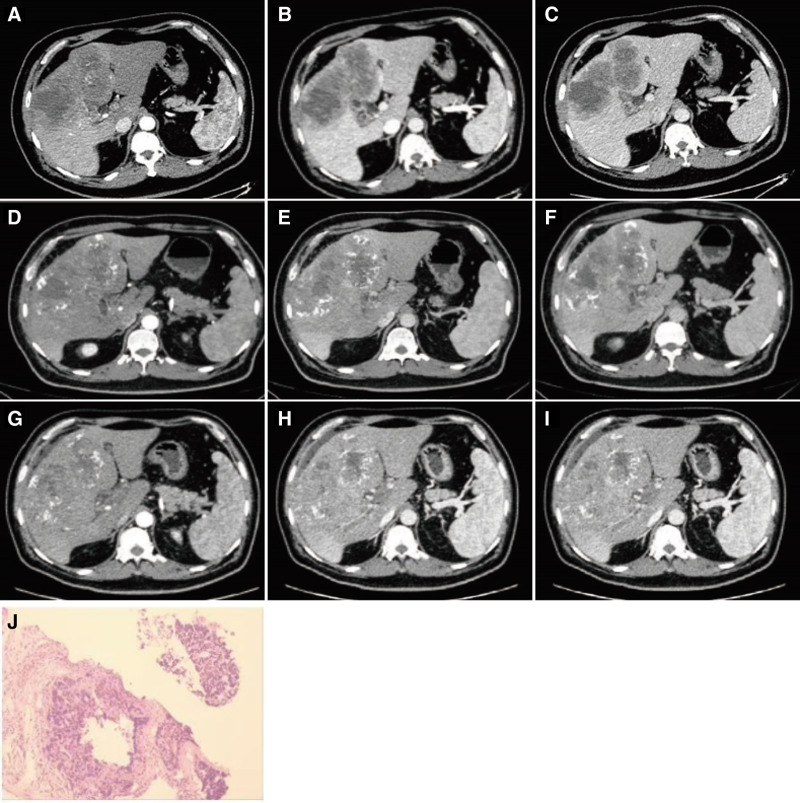
(A) The pretreatment enhanced CT arterial phase; (B) The pretreatment enhanced CT venous phase; (C) The pretreatment enhanced CT delayed phase; (D) The enhanced CT arterial phase after the first treatment; (E) The enhanced CT venous phase after the first treatment; (F) The enhanced CT delayed phase after the first treatment; (G) The enhanced CT arterial phase after the second treatment; (H) The enhanced CT venous phase after the second treatment; (I) The enhanced CT delayed phase after the second treatment; and (J) A microscopic section of the pathology. CT = computed tomography.

A 60-year-old male patient was referred to our department after a liver mass was incidentally discovered during a routine health examination. His physical examination was unremarkable, and he had no history of hepatitis or other comorbid conditions. Laboratory tests revealed an elevated alpha-fetoprotein level of 310.5 ng/mL (reference range: 0–7 ng/mL), while other tumor markers, including neuron-specific enolase, carcinoembryonic antigen (CEA), prostate-specific antigen, CA19-9, and CA72-4, were within normal limits. Contrast-enhanced abdominal computed tomography (CT) revealed a 115 × 65 mm hypervascular mass in the liver, with no abnormalities noted in the pancreas, spleen, kidneys, or adrenal glands (Fig. 1A–C). Liver biopsy confirmed neuroendocrine carcinoma (Fig. 1J). Immunohistochemical analysis showed positivity for CK, synaptophysin (Syn), chromogranin A (CgA), CD56, and INSM1, with a Ki-67 proliferation index exceeding 50%; somatostatin receptor subtype 2 was negative. Gastrointestinal endoscopy and positron emission tomography (PET)/CT excluded extrahepatic primary lesions. According to the 5th edition (2019/2022) World Health Organization (WHO) Classification of Digestive System Tumors the diagnosis of grade 3 PHNET was established.

Due to the presence of distant metastases and multiple intrahepatic lesions, the tumor was deemed unresectable. The patient underwent TACE with 9 mL of ethiodized poppyseed oil and 30 mg of doxorubicin, followed by systemic chemotherapy with the EP regimen (cisplatin and etoposide). One month later, follow-up CT showed no significant reduction in tumor size and newly developed tumor thrombus in the portal vein (Fig. 1D–F), indicating progressive disease according to response evaluation criteria in solid tumors 1.1 criteria. A second cycle of treatment was initiated, including hepatic arterial infusion chemotherapy (HAIC) using the FOLFOX regimen, repeated EP chemotherapy, and octreotide. On day 9 after the second treatment, the patient was readmitted with severe diarrhea and bone marrow suppression caused by chemotherapy. On day 38 after the second treatment, the patient again developed severe diarrhea. Follow-up CT imaging (Fig. 1G–I) revealed tumor enlargement and extensive portal vein tumor thrombosis, indicating further disease progression. Three months after the second treatment, the patient died from acute obstructive suppurative cholangitis and sepsis.

## 3. Case2

**Figure 2.**
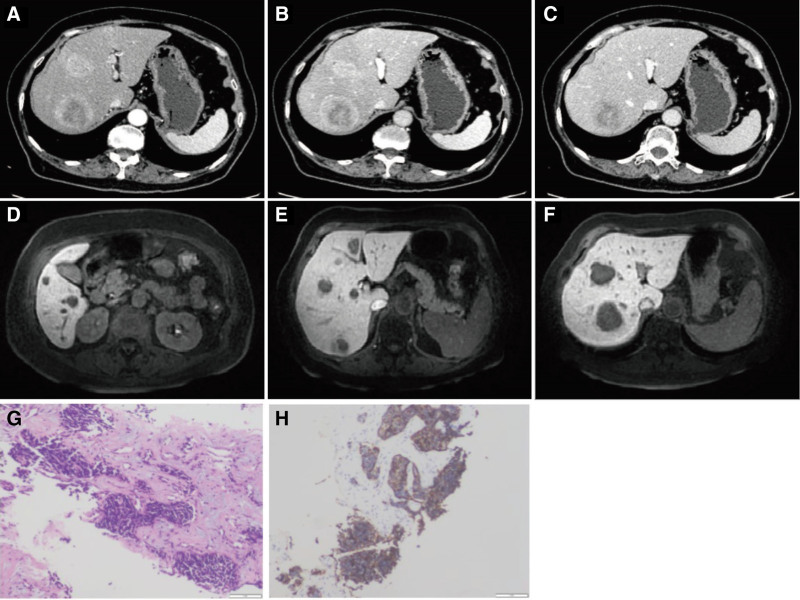
(A) The pretreatment enhanced CT arterial phase; (B) The pretreatment enhanced CT venous phase; (C) The pretreatment enhanced CT delayed phase; (D–F) MRI shows multiple lesions in the liver; (G) A microscopic section of the pathology; and (H) Positive CgA staining. CgA = chromogranin A, CT = computed tomography, MRI = magnetic resonance imaging .

A 71-year-old female patient with a history of well-controlled diabetes mellitus and no prior liver disease was admitted after routine imaging revealed multiple space-occupying lesions in the liver (Fig. 2A–C).Physical examination was unremarkable. Tumor markers, including alpha-fetoprotein, PIVKA-II, and CA19-9, were all within normal ranges. Contrast-enhanced abdominal CT and gadoxetic acid-enhanced magnetic resonance imaging (Primovist® magnetic resonance imaging) confirmed the presence of multiple hepatic masses, highly suggestive of malignancy with a possible metastatic origin (Fig. 2D–F). However, gastrointestinal endoscopy and PET/CT scans revealed no evidence of an extrahepatic primary tumor. Histopathological examination confirmed neuroendocrine carcinoma (Fig. 2G). Immunohistochemical staining showed positivity for CK, Syn, CgA, INSM1, and CD56, with a Ki-67 proliferation index of over 70% (Fig. 2H). The tumor was negative for hepatocyte markers (HC), Arginase-1, GPC-3, CK7, CK19, TTF-1, Napsin A, and LCA. A final diagnosis of primary hepatic large-cell neuroendocrine carcinoma (LCNEC, G3) was established according to the 2019/2022 WHO Classification.

Due to the multifocal nature of the lesions, curative surgery was not feasible. In light of the poor outcome observed in Case 1, the patient received a combined regimen of drug-eluting bead TACE (d-TACE), HAIC, and long-acting octreotide acetate microspheres. Long-acting octreotide acetate microspheres were included primarily for symptomatic palliation. The patient presented with severe diarrhea and vomiting, likely caused by the release of bioactive substances from tumor necrosis. Octreotide was administered to alleviate these hormone-related symptoms, in accordance with its established role in symptom control for functional neuroendocrine tumors. The d-TACE procedure employed 70 to 150 μm drug-eluting microspheres loaded with 30 mg of doxorubicin, while HAIC was administered using the FOLFOX regimen (folinic acid, 5-fluorouracil, and oxaliplatin). Continuous intravenous somatostatin infusion via micropump was used concurrently to alleviate hormone-related symptoms. A single dose of octreotide acetate microspheres was administered on the day of discharge.

**Figure 3.**
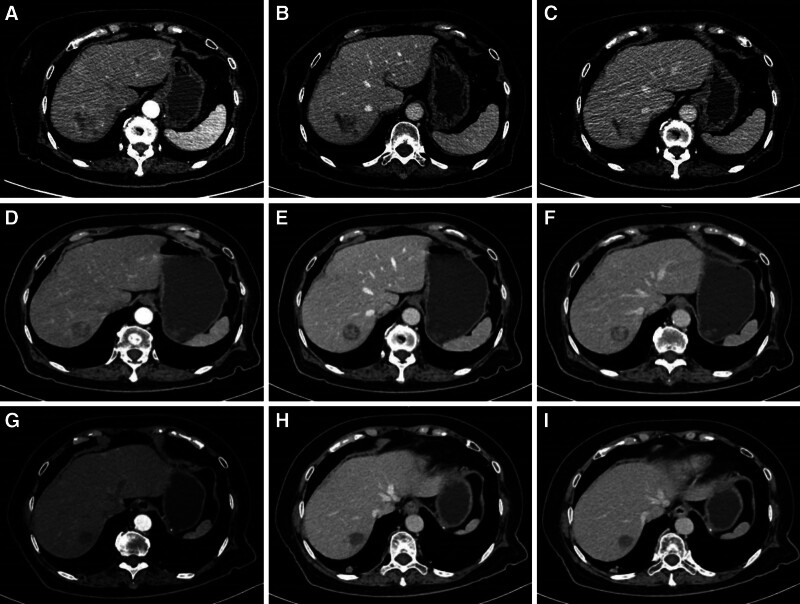
(A) The enhanced CT arterial phase after the first treatment; (B) The enhanced CT venous phase after the first treatment; (C) The enhanced CT delayed phase after the first treatment; (D) The enhanced CT arterial phase after the second treatment; (E) The enhanced CT venous phase after the second treatment; (F) The enhanced CT delayed phase after the second treatment; (G) The enhanced CT arterial phase after the third treatment; (H) The enhanced CT venous phase after the third treatment; and (I) The enhanced CT delayed phase after the third treatment. CT = computed tomography.

On day 12 post-treatment, the patient was readmitted due to severe diarrhea and vomiting. Follow-up CT imaging revealed significant tumor shrinkage, reduced enhancement, and newly developed necrosis in some lesions (Fig. 3A–C). The patient subsequently received the second and third cycles of therapy at one and 3 months after the initial treatment, respectively, following the same protocol (Fig. 3D–I). Gastrointestinal side effects were markedly milder with each successive cycle.

**Figure 4.**
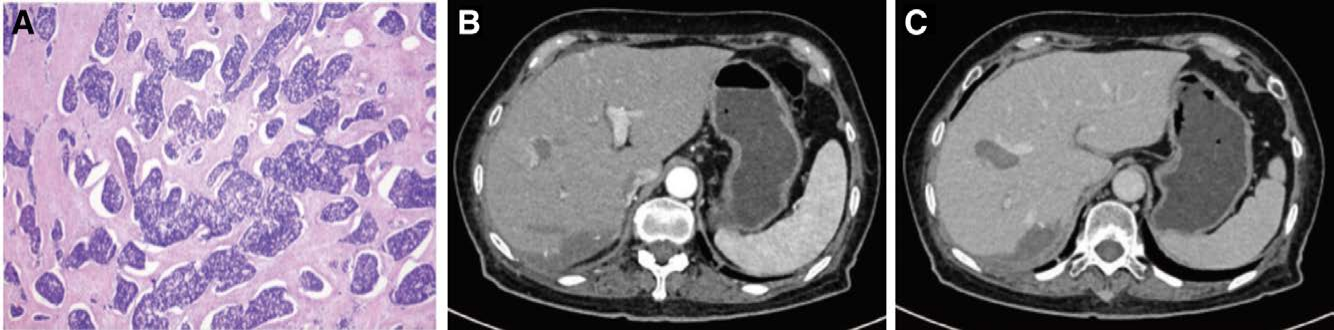
(A) Microscopic section of the postoperative pathology; (B and C) One month after surgery, follow-up CT scan showed no recurrence of the tumor. CT = computed tomography.

After 3 treatment cycles, imaging demonstrated substantial tumor regression and the disappearance of viable tumor tissue. According to response evaluation criteria in solid tumors 1.1 criteria, the response was consistent with partial remission, and the patient was considered eligible for curative surgery. Two months later, she underwent laparoscopic right posterior sectionectomy and partial left hepatectomy. The S4 lesion adjacent to the middle hepatic vein was not resected but was subsequently treated with radiofrequency ablation on postoperative day 9. Postoperative histopathology confirmed LCNEC with treatment-related changes, including necrosis and bile stasis (Fig. 4A–C). Immunohistochemistry revealed CgA(+), Syn(+), CD56(+), INSM1(+), Ki-67 (60%+), mutant P53, loss of Rb expression (Rb−), and negative somatostatin receptor subtype 2. No tumor progression or new lesions were observed at the three-month postoperative follow-up.

## 4. Discussion

Neuroendocrine tumors (NETs) most commonly arise in the gastrointestinal tract, followed by the lungs, and frequently metastasize to the liver.^[13,14]^ PHNET is exceedingly rare, and its diagnosis relies on histopathological and immunohistochemical confirmation after excluding metastatic neuroendocrine tumors.^[7]^ According to the 5th edition of the WHO Classification of Digestive System Tumors, PHNETs are categorized into low-grade (G1), intermediate-grade (G2), high-grade neuroendocrine carcinoma (G3), and mixed neuroendocrine-non-neuroendocrine neoplasms (MiNEN).^[15]^ Both diagnoses were confirmed according to the 5th edition (2019/2022) WHO Classification of Digestive System Tumors by the same pathology team at our institution. LCNEC represents a poorly differentiated, high-grade subtype of neuroendocrine carcinoma characterized by large polygonal cells, prominent nucleoli, high mitotic activity, and extensive necrosis. Compared with small-cell NEC or well-differentiated NET G3, LCNEC typically demonstrates a more aggressive clinical course, higher proliferative index, and poorer overall prognosis. Moreover, LCNEC often exhibits resistance to conventional platinum–etoposide chemotherapy, although some cases may respond to locoregional therapies such as TACE or HAIC. These pathological and biological differences highlight the importance of individualized treatment planning based on tumor histology and molecular features. Notably, the second case was classified as LCNEC. This pathological distinction may have implications for treatment response and survival. Due to its rarity, no standardized treatment guidelines currently exist. Surgical resection remains the preferred therapeutic approach; however, most patients are asymptomatic in early stages and are diagnosed at an advanced, unresectable stage. For unresectable cases, the European Neuroendocrine Tumor Society (ENETS) guidelines recommend the EP regimen (etoposide + cisplatin) as first-line chemotherapy for high-grade neuroendocrine carcinomas (NECs).^[16]^ Studies have also demonstrated that TACE effectively controls tumor growth and progression, potentially achieving complete remission and enabling surgical intervention.^[17,18]^ Additionally, somatostatin analogs have been reported not only to alleviate hormonal symptoms in functional NETs but also to delay disease progression in well-differentiated cases.^[19–21]^

**Figure 5.**
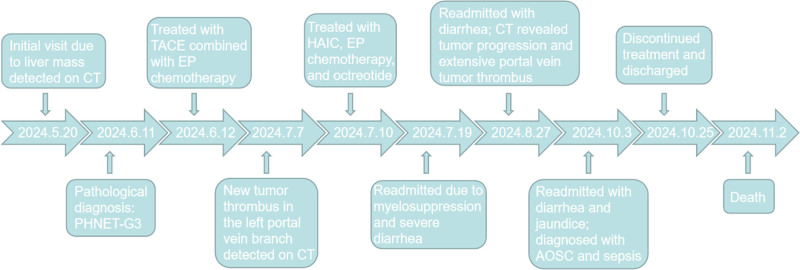
The treatment and diagnostic timeline of the Case 1.

**Figure 6.**
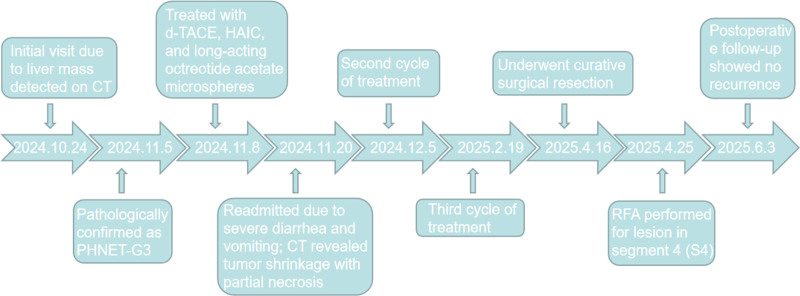
The treatment and diagnostic timeline of the Case 2.

In this study, 2 cases of PHNET were diagnosed according to the WHO Classification of Digestive System Tumors (5th edition). The first patient initially received TACE combined with the EP regimen, followed by HAIC with EP after disease progression. Despite these interventions, the tumor progressed, leading to death from acute obstructive suppurative cholangitis and sepsis. This suggests that the EP regimen may not be suitable for PHNET, though further large-scale studies are required. The second patient achieved complete remission after 3 cycles of d-TACE, HAIC, and long-acting octreotide, ultimately undergoing curative resection. This is also the first report demonstrating the favorable efficacy of HAIC treatment for PHNET. Notably, both patients developed severe gastrointestinal symptoms after the first treatment cycle, likely due to the release of bioactive substances during tumor necrosis. In the first case, persistent tumor progression correlated with recurrent gastrointestinal symptoms, whereas symptom resolution in the second case paralleled successful tumor control. Figures 5 and 6 present the treatment timelines for the 2 patients, respectively.

As a case report, this study has certain limitations. This study is limited by the small sample size (two cases), lack of a control group or standardized protocol, short follow-up duration, and absence of patient-reported outcomes. Diagnostic uncertainty cannot be fully excluded despite negative PET/CT and endoscopic examinations. Future multicenter studies with longer follow-up are warranted.

In summary, the treatment regimen used in Case 2 may be worth considering as a potential therapeutic option. The patient has remained disease-free during 3 months of postoperative follow-up, suggesting a favorable short-term response. Nevertheless, given the high-grade nature of the tumor (Ki-67 > 60%), long-term recurrence risk remains uncertain, and further follow-up is necessary to confirm the durability of this response.

## 5. Conclusion

The combination of d-TACE, HAIC, and octreotide acetate microspheres appears to be a feasible and potentially beneficial strategy for unresectable PHNET. However, larger studies are required to confirm its efficacy and long-term outcomes.

## Author contributions

**Project administration:** Xiang Lan.

**Writing – original draft:** Haiyang Hu, Kai Chen.

**Writing – review & editing:** Heng Xiao.
